# Multi-legged robots: progress and challenges

**DOI:** 10.1093/nsr/nwac214

**Published:** 2022-10-18

**Authors:** Yue Gao, Bo Su, Lei Jiang, Feng Gao

**Affiliations:** MoE Key Lab of Artificial Intelligence, AI Institute, Shanghai Jiao Tong University, China; China North Artificial Intelligence and Innovation Research Institute, China; China North Artificial Intelligence and Innovation Research Institute, China; State Key Laboratory of Mechanical System and Vibration, School of Mechanical Engineering, Shanghai Jiao Tong University, China

## Abstract

Multi-legged robots have achieved great advancements and gained attention from academia and industry. Opportunities are rising for their potential applications in human society and dangerous environments.

Considerable attention has been focused on multi-legged robots, with progress being made in the areas of mechanical design, actuators, controls, planning, and learning-based methods, as shown in Fig. 1. Legged robots are multi-input, multi-output, multi-end-effector systems. Hence, they are challenging to control when traversing unstructured environments. In recent years, technical progress has resulted in more real-world applications of legged robots.

**Figure 1.**
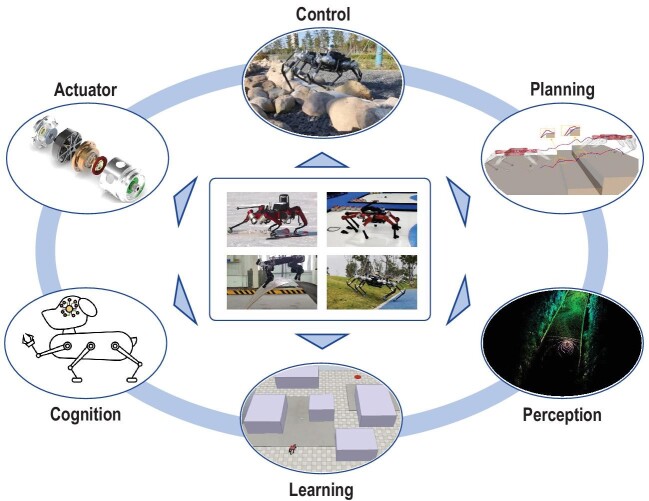
The key components of multi-legged robots.

The design of the actuator is an important factor for multi-legged robots. To meet the requirements of flexibility and the dynamics of physical interactions, the actuator's power density, compactness and impact mitigation capability are important considerations. Currently, the actuators of multi-legged robots are mainly electrical, pneumatic and hydraulic. Due to the advantages of high-power and low-cost motors, electrical actuators are commonly utilized, including stiff actuators, series elastic actuators (SEAs) and proprioceptive actuators [1,2]. Although traditional stiff actuators are widely studied, the insufficient impact mitigation capability limits the adaptability of the robots. In comparison, SEAs can reduce impacts significantly, yet are complex to model and limited by their bandwidth [1]. Due to their high performance and low cost, proprioceptive actuators are typically utilized [2]. With further improvements in the torque density, the proprioceptive actuators have a greater potential for use in legged robots.

Every legged creature in nature has acquired its unique capability to adapt to various environments. The design of bio-inspired controllers is inspired by these creatures’ motion patterns. For example, a neural oscillator is formulated to simulate the motion patterns of mammals, and realizes the limb coordination and rhythmic movement of multi-legged robots. For more robust and agile locomotion control, model-based controllers with kinematic and dynamic constraints are formulated to guarantee physical feasibility [3].

Planning serves as a connecting link between the perception and control modules. Methods such as differential dynamic programming and direct collocation are commonly utilized. However, due to high non-linearity and limited on-board computation resources, these methods have limited application in legged robots in real time. One approach to reduce the computing complexity is to design a hierarchical planner, with the top planner learning the global semantics or capability of the legged robots while the bottom planner solves the local motion trajectory. Another solution is to design an offline and online framework by combining trajectory optimization and model predictive control [4]. The key challenge for the planning of legged robots is finding a balance between the computational complexity and an accurate trajectory that matches the robots’ motion ability. Hence, advanced learning and optimization methods are all potential directions to explore.

On legged robots, proprioceptive and exteroceptive sensors are necessary for the perception of the surroundings in the physical world, including IMUs, torque sensors, force sensors, lidar and cameras. For mapping and localization, the well-established methods in wheeled robotics could be utilized for legged robots. However, to adapt to unstructured environments, dense information of the terrain is often required. With the popularization of high-precision sensors, such as lidar and RGB-D cameras, a local elevation map and traversability map can be constructed [5,6]. These maps can be further utilized in the planning module. With the advancement of perception, more potential uses of multi-legged robots could be explored with an improved semantic understanding of the environment.

Recent developments in the field of artificial intelligence (AI) have resulted in new opportunities for legged robots and have improved the coexistence and cooperation between legged robots and humans. Except for the direct deployment of general algorithms in AI, methods designed from the perspective of legged robotics have shown great potential to overcome some of the limitations in the field. Recent works have demonstrated that the high-dimensional complex dynamics of actuators and robotics can be modeled through deep neural networks, resulting in the learning of agile locomotion skills. Self-training in simulators with sim-to-real transfer learning is a new trend in legged robotics that can avoid excessive hardware consumption and experimental risks. Deep reinforcement learning and supervised learning methods have outperformed traditional approaches in actuator and locomotion control [7], and enable legged robots to autonomously traverse challenging natural terrains. In the future, data-efficient and generalized learning methods are needed for legged robots to accomplish more challenging tasks.

The latest advancements in neuroscience and cognitive science provide inspiration for the concepts of embodied and evolutionary robotics, in the field of legged robotics. Recent studies achieved behavior and motion intelligence through the harmony of software and hardware. In addition, the abilities of active adaptation and lifelong learning are beginning to emerge [8]. Although it cannot yet be called ‘artificial consciousness’, these abilities will, in the future, effectively enable legged robots to adapt to environments over their entire existence.

Despite progress in mechanical design, actuators, controls, planning and perception, multi-legged robots still face technical and application challenges. Compared to humans, multi-legged robots are not as powerful, flexible and reliable due to a lack of powerful actuators and high-level autonomy, especially when faced with non-rigid terrains and unstructured environments. However, humans need, and expect to utilize, multi-legged robots in extreme and dangerous environments, such as in space and nuclear facilities. In recent applications, multi-legged robots were deployed in industrial scenarios for patrol and inspection, such as Spot from Boston Dynamics and ANYmal from ANYbotics. During the Beijing 2022 Olympic Winter Games, skiing, skating and curling hexapod robots debuted. Faced with the challenges of a complex natural ice and snow environment, skiing and skating legged robots exhibited the potential for application in sports and in the exploration of polar regions [9]. In the future, the study of human–robot interactions and guaranteed safety control is of great importance to ensure multi-legged robots’ broader application in daily life.

In conclusion, there are significant challenges and research opportunities for multi-legged robots. With developments in mechanical engineering, control theory and AI, multi-legged robots will further enhance the coexisting-cooperative-cognitive approach to human–robot interactions, allowing legged robots to be further involved in various aspects of human life.
